# Very late-onset neuromyelitis optica spectrum disorder beyond the age of 75

**DOI:** 10.1007/s00415-015-7766-8

**Published:** 2015-05-10

**Authors:** Markus Krumbholz, Ulrich Hofstadt-van Oy, Klemens Angstwurm, Ingo Kleiter, Sven Jarius, Friedemann Paul, Orhan Aktas, Grete Buchholz, Peter Kern, Andreas Straube, Tania Kümpfel

**Affiliations:** Institute of Clinical Neuroimmunology, Ludwig Maximilian University, Max-Lebsche-Platz 31, 81377 Munich, Germany; Klinik für Neurologie, Klinikum Bayreuth—Klinik Hohe Warte, Bayreuth, Germany; Department of Neurology, University of Regensburg, Regensburg, Germany; Department of Neurology, St. Josef-Hospital, Ruhr-University, Bochum, Germany; Molecular Neuroimmunology Group, Dpt. of Neurology, University of Heidelberg, Heidelberg, Germany; NeuroCure Clinical Research Center and Department of Neurology, Charité University Medicine Berlin, Berlin, Germany; Department of Neurology, Medical Faculty, Heinrich Heine University, Düsseldorf, Germany; Department of Neurology, Ludwig Maximilian University, Munich, Germany; Klinik für Neurologie und Klinische Neurophysiologie, Asklepios Fachklinikum, Teupitz, Germany

**Keywords:** Neuromyelitis optica (Devic syndrome), Myelitis, Aquaporin 4 antibodies, Very late-onset, Elderly/old-age, Therapy/immunosuppression

## Abstract

Aquaporin-4 antibody (AQP4-Ab)-positive neuromyelitis optica spectrum disorder (NMOSD) is a rare but often severe autoimmune disease with median onset around 40 years of age. We report characteristics of three very-late-onset NMOSD (including complete NMO) patients >75 years of age, in whom this diagnosis initially seemed unlikely because of their age and age-associated concomitant diseases, and briefly review the literature. All three patients, aged 79, 82 and 88 years, presented with a spinal cord syndrome as the first clinical manifestation of AQP4-Ab-positive NMOSD. They all had severe relapses unless immunosuppressive therapy was initiated, and one untreated patient died of a fatal NMOSD course. Two patients developed side effects of immunosuppression. We conclude that a first manifestation of NMOSD should be considered even in patients beyond the age of 75 years with a compatible syndrome, especially longitudinally extensive myelitis. Early diagnosis and treatment are feasible and highly relevant. Special attention is warranted in the elderly to recognize adverse effects of immunosuppressive therapies as early as possible.

## Background

Neuromyelitis optica (NMO) is a rare but often severely disabling autoimmune disease of the central nervous system affecting predominantly women, most frequently in their 30s to 40s [1, 2]. Aquaporin-4 antibodies (AQP4-Ab) are present in about 80 % of NMO patients and in a subset of patients with isolated longitudinally extensive myelitis or isolated optic neuritis, who are then considered to have formes frustes of NMO. NMO and its incomplete forms are referred to as NMO spectrum disorders (NMOSD) [3]. AQP4-Ab are highly specific for NMOSD, even in the elderly [4].

## Case series

### Case 1

A 79-year-old man was admitted to a municipal hospital with a bilateral sensory level below T3 and gait ataxia since the last month (EDSS 6.5). Magnetic resonance imaging (MRI) revealed spinal cord lesions at vertebra C1-4, at T3/4, and brain microangiopathy. Cerebrospinal fluid (CSF) analysis showed mildly increased albumin, but normal immunoglobulin G (IgG), negative oligoclonal bands (OCB), and a normal cell count. Autoimmune myelitis was suspected, and symptoms improved after intravenous glucocorticoids (EDSS 5.0).

Four months later, a second attack occurred with severe left-sided optic neuritis and worsening of sensorimotor symptoms (EDSS 9.0). MRI revealed an enlarged myelon lesion (medulla oblongata to vertebra C5, partially gadolinium-enhancing). CSF analysis showed pleocytosis (32/µl). Symptoms improved after intravenous steroids (EDSS 8.0).

**Fig. 1:**
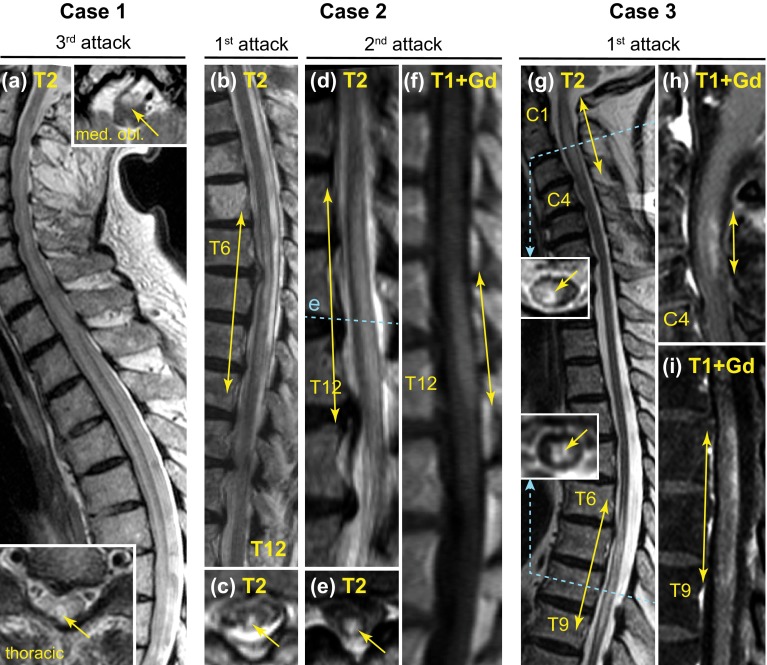
MRI scans of case 1–3 showing longitudinally extensive spinal cord lesions.* Yellow solid arrows* indicate extensions of lesions. The lesion extended throughout the entire myelon and there were also lesions in the brainstem in case 1 (**a**) (*upper inset* in **a**). *Blue dashed lines* in the sagittal images indicate the levels of related axial scans. *med. obl.* axial scan at level of medulla oblongata, *C* and *T* indicate the respective cervical and thoracic vertebrae; *T2* T2 weighed MRI sequence, *T1* T1 weighed MRI sequence, +*Gd* gadolinium-enhanced sequence

After another month, a third attack occurred with dysphagia, a high-cervical transverse spinal cord syndrome with marked tetraparesis, and another severe optic neuritis (blindness in left eye; EDSS 9.0). He was admitted to a university hospital. MRI revealed a lesion extending over the entire myelon and brainstem involvement (Fig. 1a). CSF analysis showed 43 cells/µl including granulocytes. AQP4-Ab were positive in serum and CSF (ΔMFI 816 and 181, respectively, FACS-based assay), and diagnosis of AQP4-Ab positive NMO [5] as part of NMOSD [3] was established. Intravenous steroids were given, followed by plasma exchange, without improvement. After further deterioration with respiratory insufficiency because of high cervical myelon involvement (EDSS 9.5) and pneumonia, he required intensive care and died shortly thereafter.

### Case 2

An 88-year-old woman experienced numbness in her legs and moderate paraparesis with impaired gait since 2 days. Spinal MRI demonstrated a myelon lesion from vertebra T6-9 (Fig. 1b-c), which was initially attributed to compression myelopathy because of concomitant vertebral disc protrusions. Without specific treatment, she recovered partially and was able to walk with a crutch for >100 m (EDSS 6.0). Her previous medical history was negative for prior potential attacks, but included a transient ischemic attack with dysarthria for <24 h 1.5 years before; cerebral MRI had not shown inflammatory lesions.

Eight months later, she was re-admitted with an anew gait impairment and sensorimotor paraparesis (MRC grade 2–3, EDSS 8.5). MRI demonstrated a new T2 hyperintense myelon lesion from vertebra T10–T12 with central gadolinium enhancement (Fig. 1d–f). Visual evoked potentials had low amplitudes bilaterally and normal latencies. CSF showed mild lymphomonocytic pleocytosis and positive OCB. Serum AQP4-Ab were positive (1:3200, cell-based immunofluorescence assay), as were antibodies against dsDNA and cardiolipin. Diagnosis of AQP4-Ab positive NMOSD was established, and the first myelon lesion was retrospectively attributed also to NMOSD. Treatment included methylprednisolone 5 × 1 g, a second cycle of 5 × 2 g, and then plasma exchange. She recovered partially (EDSS 7.0). Azathioprine was given up to 150 mg/d (2.2 mg/kg); thiopurine S-methyltransferase activity was normal. After 5 months of therapy, regular blood testing revealed pancytopenia. Azathioprine was stopped, but thrombocytopenia persisted and she died of intestinal bleeding. In addition to azathioprine as a likely cause for bone marrow suppression and thrombocytopenia, she had also developed anti-platelet antibodies.

### Case 3

A woman was admitted shortly before her 83rd birthday with numbness and weakness in her right arm, impaired sensation below T10 bilaterally, and high-graded paraparesis since 2 days (EDSS 8.0). Her previous medical history and family history was unremarkable, in particular, for previous attack-like clinical events or immunological disease. Infectious myelitis was suspected, and antimicrobial treatment started.

MRI demonstrated two longitudinally extensive myelon lesions (foramen magnum to vertebra C4, T6-9), both with dorsal gadolinium enhancement (Fig. 1g–i), but no inflammatory brain lesions. CSF analysis showed mild pleocytosis (10 cells/µl, 3 % neutrophils) with one CSF-restricted band, negative MRZ reaction, and normal IgG and albumin ratios. An extensive search for microbial pathogens in serum and CSF was negative. She reported no visual symptoms, but visual evoked potentials demonstrated delayed P100 latencies bilaterally with normal amplitudes. Screening for rheumatic disease showed high titers for antinuclear antibodies (1:12,800, negative for standard ENA panel) without further clinical or laboratory evidence of rheumatologic disease.

Autoimmune myelitis being suspected, she received methylprednisolone (5 × 500 mg i.v.). Serum AQP4-Ab turned out positive (1:320, immunofluorescence assay), and diagnosis of AQP4-Ab positive NMOSD was established. Since there was no improvement and the patient refused plasma exchange, she received a second cycle of methylprednisolone (5 × 2 g i.v. with oral taper), and azathioprine was started (up to 125 mg/d). She improved continuously and was able to walk with a walking frame and lived independently again (EDSS 6.5).

Three months later, she developed cytomegalovirus pneumonia and hepatopathy, probably related to azathioprine. At the time of admission, she had normal leukocyte counts and moderate lymphopenia (11 % ≙ 570/µl). Azathioprine was discontinued. She recovered completely after receiving ganciclovir. Immunosuppression was switched to mycophenolate mofetil which is well tolerated (1.5 g/d). Until now, she has remained relapse-free for 2 years.

## Conclusions

According to independent cohorts, the mean onset of NMO is around 40 years [1, 2]. We report three patients who were much older at the time of first manifestation, so that initially NMOSD was considered unlikely. Patients with very late-onset NMOSD (>75 years) have hitherto only rarely been reported in detail, and case 2 is, to our knowledge, the oldest patient described so far (Table 1).

**Table 1:** Screening Pubmed for NMOSD cohorts and case reports with at least 1 patient with onset >60 years did not reveal patients at least as old as our NMOSD patient with very late-onset at 88 years. Onset >50 years is usually defined already as late-onset

Location/ethnicity (age limit for study inclusion)	Number of patients	Max. onset (age in years)	Clinical characteristics	References
Korea (–)	92	63	Patients with onset >50 year (22 %): more often myelitis onset and higher ARR	[7]
France, 87 % Caucasian (–)	125	66	20 % onset >50 year	[12]
Anglo-Saxon Americans and Hispanic Americans (–)	8	73	Oldest patient with ON onset. No LTI, relapse after 4 months, fatal course	[13] (before AQP4-Ab)
Australia (–)	71	79.6	Patients with onset >50 year: less often optic neuritis onset	[9]
Europe, 93 % Caucasian (late-onset NMOSD >50 year)	108	82.5	Myelitis onset in 67 %. Mean follow-up 4.6 year, 82 % with relapses	[6]
Japan (–)	583	86	Patients with onset >60 year: more often myelitis onset	[8]
Italy (late-onset case report)	1	64	Optic neuritis and myelitis onset. Two relapses within months, fatal course	[14] (before AQP4-Ab)
USA (late-onset case report)	1	69	Optic neuritis and myelitis onset	[15] (before AQP4-Ab)
France (late-onset case report)	1	77	Myelitis onset. W/o LTI, relapse after 1 year	[16]
USA (late-onset case report)	1	81	Brainstem and myelitis onset. No LTI, several relapses and death within about 1 year	[17] (before AQP4-Ab)
USA (late-onset case report)	1	85	Myelitis onset, no long-term follow-up	[18]

*ARR* annualized relapse rate, *before AQP4-Ab* study performed before availability of AQP4-Ab testing, *LTI* long-term immuno-suppressive therapy

Of note, all our patients initially presented with myelitis. More frequent myelitis (vs. optic neuritis) as initial presentation is consistent with recent reports for patients with late-onset (>50–60 years) from Europe, USA and Japan [6–9] (see also Table 1). Interestingly, also multiple sclerosis late-onset patients had more often spinal cord lesions compared to young-onset (<50 years) patients [10].

Both patients (case 1 and 2) who did not receive immunosuppression after their first attack experienced one or more subsequent relapses shortly afterwards, and case 1 who never received immunosuppression had a fulminant course and died of NMO sequelae within 1 year from disease onset. An older age at onset was associated with earlier death due to myelitis and infection [6]. Unlike in multiple sclerosis, relapse activity in NMO does not seem to decrease with age; even very old patients are at risk of further disabling attacks (our cases, ref [11], and Table 1). This suggests that also patients with late-onset need long-term prophylactic treatment to prevent subsequent relapses.

The benefits of an immunosuppressive therapy have to be weighed against an increased risk of adverse effects in the elderly. Clearly, attention and alertness is warranted especially in older individuals to recognize adverse effects as early as possible. Further NMOSD treatment studies should pay special attention to patients with late-onset.

In all three cases, NMOSD initially was not considered as the first-line diagnosis, mainly because of the old age and past medical history. In this age group, seemingly competing explanations of myelon lesions are common, including vascular, infectious, and orthopedic causes, as initially falsely suspected also here. However, delayed diagnosis and treatment can lead to subsequent relapses and fatal outcome. Therefore, it is mandatory to consider NMOSD irrespective of age and past medical history in patients with a compatible syndrome, especially longitudinally extensive myelitis. The diagnostic workup should include MRI, CSF investigations including differential cell count, serum AQP4-Ab testing using recombinant cell-based assays, and electrophysiology to detect and treat NMOSD as early as possible.

## Appendix

Members of the Neuromyelitis Optica Study Group (NEMOS) in alphabetical order: P. Albrecht, University of Düsseldorf; O. Aktas, University of Düsseldorf; K. Angstwurm, University of Regensburg; A. Berthele, Technische University of München; N. Borisow, Charite Berlin; T. Böttcher, Bonhoeffer Klinikum Neubrandenburg; J. Brettschneider, University of Ulm; B. Ettrich, University of Leipzig; J. Faiss, Asklepios Klinik Teupitz; A. Gass, University Hospital Mannheim; C. Geis, University of Würzburg; K. Guthke, Klinikum Görlitz; H-P. Hartung, University of Düsseldorf, K. Hellwig, Ruhr-University Bochum; B. Hemmer, Technical University Munich; F. Hoffmann, Krankenhaus Martha-Maria Halle; M. Kaste, Nordwest-Krankenhaus Sanderbusch, Sande; U. Hofstadt-van Oy, Klinikum Bayreuth; S. Jarius, University of Heidelberg; P. Kermer, Nordwest-Krankenhaus Sanderbusch, Sande; P. Kern, Asklepios Klinik Teupitz; C. Kleinschnitz, University of Würzburg; I. Kleiter, Ruhr-University Bochum; W. Köhler, Fachkrankenhaus Hubertusburg; E. Kolesilova, Asklepios Klinik Teupitz; M. Krumbholz, Ludwig Maximilians University Munich; T. Kümpfel, Ludwig Maximilians University Munich; S. Langel, Landeskrankenhaus Rheinhessen; F. Lauda, University of Ulm; M. Liebetrau, Evangelische Bathildiskrankenhaus Bad Pyrmont gGmbH; R. Linker, University of Erlangen; W. Marouf, Heliosklinik Stralsund; M. Marziniak, Isar-Amper Klinik Ost Munich; I. Metz, University of Göttingen; C. Mayer, University of Frankfurt; A. Melms, University of Erlangen; C. Münch, Charité University Medicine Berlin; O. Neuhaus, Kreiskrankenhaus Sigmaringen; S. Niehaus, Klinikum Dortmund; F. Pache, Charité University Medicine Berlin; F. Paul, Charité University Medicine Berlin, H. Pellkofer, University of Göttingen; R. Reuss, Bezirkskrankenhaus Bayreuth; A. Riedlinger, Asklepios Klinik Teupitz; M. Ringelstein, University of Düsseldorf; S.P. Rommer, University of Rostock; K. Ruprecht, Charité University Medicine Berlin; S. Schippling, University of Zürich (Switzerland); S. Schuster, University of Tübingen; M. Schwab, University of Jena; M. Stangel, Medizinische Hochschule Hannover, J. Stellmann, University of Hamburg; F. Then-Bergh, University of Leipzig; C. Trebst, Medizinische Hochschule Hannover; H. Tumani, University of Ulm; C. Veauthier, Heliosklinik Stralsund; KP. Wandinger, University of Schleswig–Holstein; R. Weissert, University of Regensburg; B. Wildemann, University of Heidelberg; C. Wilke, Nervenzentrum Potsdam; A. Winkelmann, University of Rostock; L. Zeltner, University of Tübingen; C. Zentner, Martha Maria, University of Halle; U. Zettl, University of Rostock; U. Ziemann, University of Tübingen.

## Abbreviations

AQP4: Aquaporin 4
AQP4-Ab: Aquaporin-4 antibodies
CSF: Cerebrospinal fluid
EDSS: Expanded disability status scale (0–10 points, 0 = normal, 10 = death due to MS)
IgG: Immunoglobulin G
MRC: Medical Research Council (scale for muscle strength 0–5, 5 = full strength)
MRI: Magnetic resonance imaging
NEMOS: Neuromyelitis Optica Study Group
NMO: Neuromyelitis optica
NMOSD: Neuromyelitis optica spectrum disorder
OCB: Oligoclonal bands
